# Clinical and genetic characterization of a Chanarin Dorfman Syndrome patient born to diseased parents

**DOI:** 10.1186/s12881-018-0610-0

**Published:** 2018-05-29

**Authors:** Murat Durdu, Sara Missaglia, Laura Moro, Daniela Tavian

**Affiliations:** 1 Baskent University Faculty of Medicine, Department of Dermatology, Adana Hospital, Adana, Turkey; 2 Laboratory of Cellular Biochemistry and Molecular Biology-CRIBENS, Catholic University of the Sacred Heart, pz Buonarroti 30, 20145 Milan, Italy; 3 Department of Psychology, Catholic University of the Sacred Heart, Largo Gemelli 1, 20123 Milan, Italy; 4 Department of Pharmaceutical Sciences, University of Piemonte Orientale, Lgo Donegani 2, 28100 Novara, Italy

**Keywords:** Chanarin-Dorfman Syndrome, Ichthyosis, Lipid disorder, Liver involvement, Myopathy

## Abstract

**Background:**

Chanarin Dorfman Syndrome (CDS) is a rare autosomal recessive disorder characterized by ichthyosiform non-bullous erythroderma and variable involvement of the liver and the neuromuscular system. In CDS patients, the accumulation of neutral lipids inside cytoplasmic lipid droplets has been demonstrated in different tissues. To date, ninety families with this disease have been described worldwide; most of them are from Mediterranean countries.

**Case presentation:**

In this report, we describe a consanguineous Turkish family with typical features of CDS. The parents are first cousins and are both diseased. At the age of eight, their child presented CDS with non-bullous congenital ichthyosiform erythroderma, hepatosteatosis, hepatomegaly and ectropion. Electromyographic examination is compatible with myopathy. A five-year-old cousin of the child is also affected by CDS. She was born to non-affected consanguineous parents. Mutation analysis of the *ABHD5* gene revealed the previously reported mutation, N209X, which is the most frequent in Turkish patients. Lipid vacuoles, also known as Jordan’s anomaly, are detectable in their leucocytes.

**Conclusions:**

To the best of our knowledge, this is the first report of a CDS family in which both parents and their child are affected by CDS. To date, the child does not present a more severe clinical phenotype compared with those of his relatives or other CDS patients of the same age. These findings suggest that high levels of triacylglycerol accumulation, that may be supposed to be present in high amount inside the ooplasm, did not affect embryo development and foetal growth.

**Electronic supplementary material:**

The online version of this article (10.1186/s12881-018-0610-0) contains supplementary material, which is available to authorized users.

## Background

Neutral lipid storage disease with ichthyosis, which is also known as Chanarin Dorfman Syndrome (CDS; MIM 275630), is a rare autosomal recessive disease characterized by the intracellular accumulation of triacylglycerol (TG) in numerous tissues [1, 2]. The clinical phenotype involves multiple organs and systems, including skin, liver, skeletal muscle, eyes, ears, and the central nervous system. However, in this syndrome, the degree of systemic involvement is quite variable [2–5]. ABHD5 (α/β hydrolase domain 5), a cofactor for adipose triglyceride lipase (ATGL), has been identified as a causative gene of CDS [6]. Indeed, different ABHD5 mutations determine the partial or total loss of ATGL activation, leading to the accumulation of TG inside lipid droplets [2–5]. Lipid droplets are highly dynamic and ubiquitous cellular organelles. These droplets are a fundamental component of lipid homeostasis, i.e., a universal feature of eukaryotic cells that can ensure a rapidly mobilized lipid source for numerous biochemical processes [7]. Neutral lipids also accumulate in oocytes and blastomeres as lipid droplets, providing energy for mammalian embryo preimplantation, proper growth and development [8].

In the peripheral leukocytes of CDS patients, neutral lipids inside lipid droplets are easily detectable with standard or specific stains, and they are known as Jordan’s anomalies, the most common laboratory findings of the disease [9].

One hundred and twenty-eight CDS patients have been reported worldwide (Additional file 1: Table S1). For 85 of these patients, clinical diagnosis has been confirmed by *ABHD5* mutation analysis. The highest number of CDS patients (27 cases) has been described in Turkey, mainly due to the high incidence of consanguineous marriages [10]. Nevertheless, the clinical and genetic description of families in which both parents and children are affected by CDS has never been reported before.

## Case presentation

**Fig. 1:**
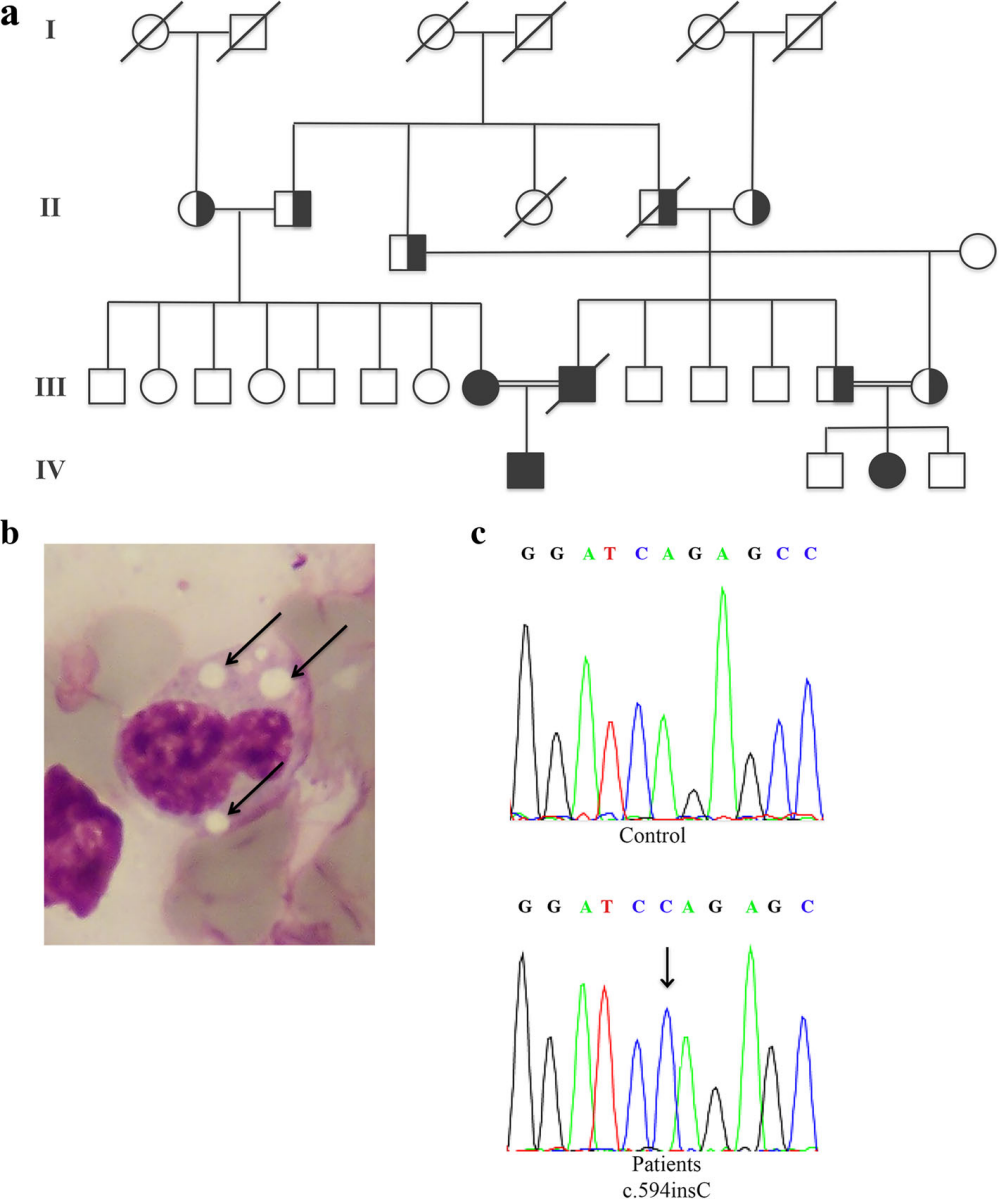
Pedigree of CDS family (**a**). Microphotographs of May-Grünwald-Giemsa buffy coats of patient III-1 showing Jordan’s anomaly (arrows); original magnification 1000× (**b**). Sequence analysis showing the c594insC (N209X) ABHD5 mutation identified in homozygous status in all family members affected by CDS (**c**)

Here, we present a CDS Turkish family with four affected members (Fig. 1a, III-1, III-2, IV-1, IV-2). The child (IV-1) was an 8-year-old male born at 38 weeks of gestation and weighing 3400 g. He presented lamellar ichthyosis at birth. At first examination (at the age of 5), he had lagophthalmos and ectropion. Ophthalmologists did not record lagophthalmos in subsequent examinations, so the condition was considered a compliance problem. He had elevated transaminase (ALT: 75 U/L; normal 5-35 U/L; GGT 31 U/L; normal 5-17 U/L), creatine phosphokinase (CK: 1677 U/L; normal 22-200 U/L), triglyceride (460 mg/dl; normal 35-130 mg/dl), and total cholesterol levels (135 mg/dl; normal 110-200 mg/dl) (Table 1). Systemic examination revealed 2 cm hepatomegaly. Abdominal ultrasonography was compatible with hepatosteatosis and hepatomegaly. Electromyographic examination revealed signs of myopathy.

**Table 1:** Clinical and laboratory features of patients with Chanarin-Dorfman syndrome

Clinical feature	Child	Father	Mother	Cousin
Ichthyosis	+	+	+	+
Leukocyte vacuoles	+	+	+	+
Creatinine phosphokinase levels (U/L)	1677	290	219	1145
Electromyographic examination	Myopathy	Normal	Normal	Myopathy
Triglyceride level (mg/dL)	460	115	74	74
Total cholesterol level (mg/dL)	135	151	115	140
Cataracts	–	–	–	–
Ectropion	+	+	+	–
Lagophthalmos	+	–	–	–
Strabism	–	–	–	–
Myopia	–	+	–	–
Hearing loss	–	–	–	–
Mental retardation	–	–	–	–
Hepatomegaly	+ (2 cm)	+	+	+ (2 cm)
Hepatosteatosis	+	+	+	+
Microcephaly	–	–	–	–
Intestinal involvement	–	–	–	–

**Fig. 2:**
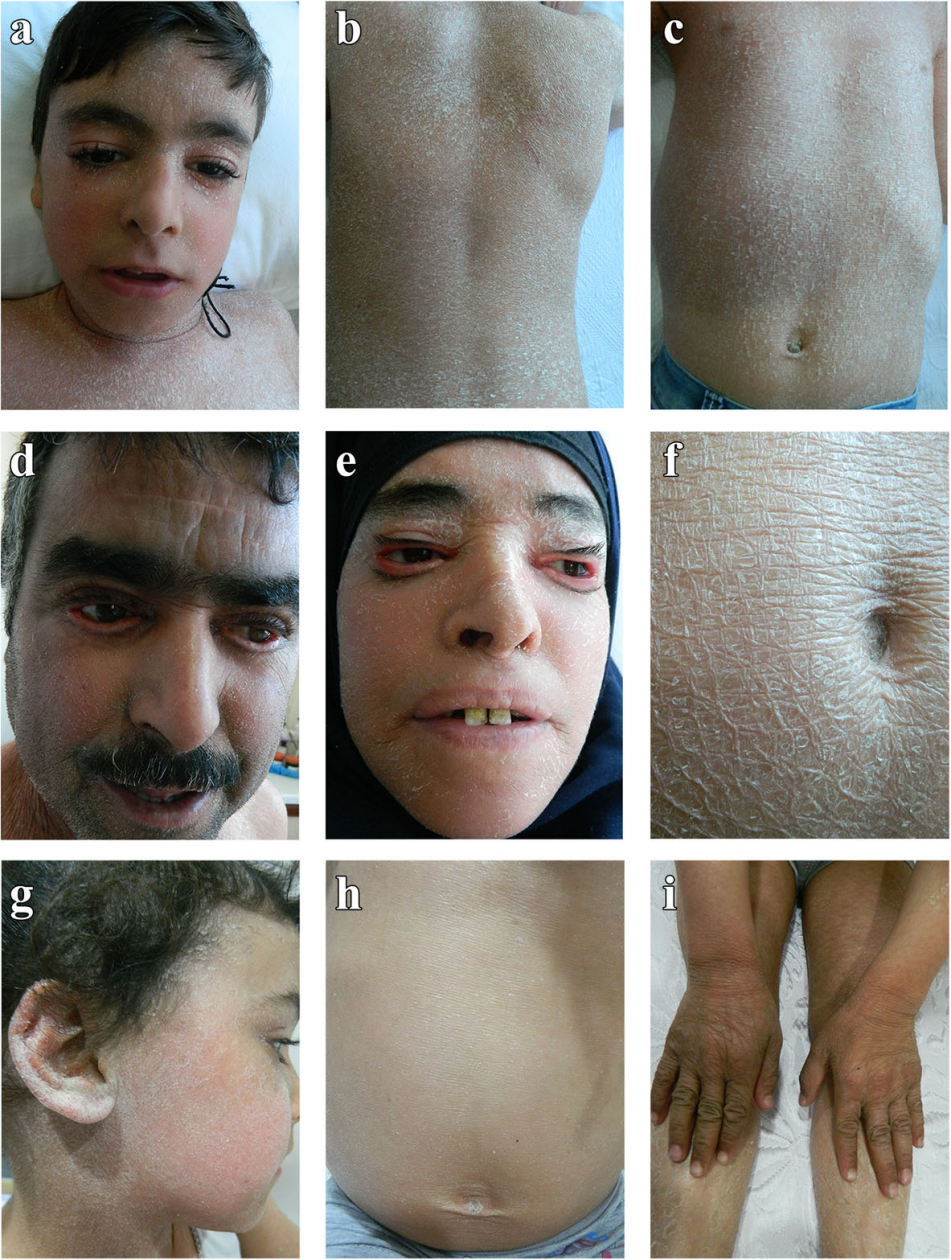
Dermatological characterization of CDS patients. Lamellar ichthyosis affecting facial region (**a**, **d**, **e**, **g**), trunk (**b**, **c**, **f**, **h**), and extremities (**i**) of child (patient IV-1; **a**, **b**, **c**), father (patient III-2; **d**), mother (patient III-1; **e**, **f**) and cousin (patient IV-2; **g**, **h**, **i**). Prominent eyelid ectropion in father (**d**) and mother (**e**)

His parents (both 44 years old) were paternal cousins. Both presented lamellar ichthyosis, ectropion, hypertriglyceridemia, hepatomegaly and hepatosteatosis (Table 1). In both parents and the child, dermatological examination revealed widespread ichthyosis on the facial region, trunk, extensor and flexural regions and scalp. The individual scales over the trunk were white, fine, translucent and semi-adherent, whereas those on the limbs and face were grey-brown, larger in size, polygonal and adherent (Fig. 2). No bullous lesions or erosions were noted. The involvement of palms and soles, dental anomaly and nail dystrophy was absent.

In the 5-year-old girl (IV-2), ichthyosis was also present in flexural regions (Fig. 2). Corrugated appearance and hyperpigmented scales were detected on foot, back, knee, and elbow. Laboratory investigation revealed increased ALT (86 U/L) (alanine transaminase), AST (aspartate transaminase) (88 U/L) and CK (1145 U/L) levels (Table 1). Electromyographic examination revealed myopathy. Abdominal ultrasonography revealed hepatosteatosis causing hepatomegaly. Ophthalmological and audiological examinations were normal.

A peripheral blood smear stained with May-Grünwald-Giemsa revealed lipid vacuoles (Jordan’s anomaly) in leucocytes from all patients (Fig. 1b). After obtaining informed consent and in accordance with the Declaration of Helsinki principles, the coding regions of the *ABHD5* gene were sequenced, and a homozygous N209X mutation was identified (Fig. 1c). For molecular analysis, oligonucleotides were selected to amplify and sequence the seven exons of *ABHD5*, their intron/exon boundaries, and the candidate promoter regions. The primer sequences and the amplification conditions were previously reported [2]. A few days after molecular diagnosis, the father died of a heart attack at the age of 44. He had no prior cardiac history. However, *PNPLA2* gene analysis was performed, excluding disease-causing mutations.

The CARE guidelines were followed in this case.

## Discussion and conclusions

The N209X mutation identified in our CDS family is the most common in the Turkish CDS population. Nur et al. compared clinical findings between patients carrying this mutation with other *ABHD5* variations and noted no significant differences [10]. In CDS patients, skin involvement is prevalent and a consistently observed clinical feature, consisting of a non-bullous congenital ichthyosiform erythroderma. Sano et al. demonstrated that the severity of ichthyosis positively correlates with TG level in the scales from patients [11]. In our affected subjects, a widespread lamellar ichthyosis detected since birth revealed a homogeneous CDS phenotype. Notably, skin involvement did not appear more severe in the child; however, it is possible to hypothesize increased accumulation of TG in ectodermal cells during embryonic development. Liver abnormalities can occur in greater than 80% of patients, ranging from hepatomegaly or liver steatosis to cirrhosis, and can be observed in young children, as occurred in this family [4, 6]. Neurological impairment was not detected in our patients; however, intellectual disability has been reported in 20% of CDS subjects and in approximately 40% of those carrying the N209X mutation [10–12]. Sensorineural hearing loss was not present in our family; however, this condition occurs in 30% of CDS patients. Muscle involvement was evident only in the two children. Clinically, the children did not exhibit significant muscle weakness, fatigue or exercise intolerance. However, an electromyographic examination (EMG) was performed because of an elevation in CK levels and revealed a myopathic pattern. In CDS, muscle abnormalities have been detected in 40% of subjects. Myopathy typically begins in the thirties, but it has also been described in very young children [2, 13].

In conclusion, we describe a family in which all members are affected by CDS. When the child was born, his mother was 36 years old. She did not indicate any infertility problems or history of abortion. This paper describes the unique reported case of natural conception and pregnancy between two CDS patients. No particular pregnancy complications were observed. Therefore, the storage of neutral lipids inside lipid droplets did not dramatically increase during oocyte maturation. Indeed, embryo development and foetal growth were not affected. To date, the child does not present new symptoms or an exacerbation of the clinical phenotype even with regard to dermatologic manifestations compared with the symptoms and phenotypes of his relatives or other CDS patients. Instead, a relevant aspect for these three patients seems to be represented by the psychogenic stress due to isolation from society, colleagues, school friends and relatives. Finally, after molecular diagnosis, the two children immediately started a special diet, poor in fatty acids with medium chain triglycerides (MCT), as hepatic and dermatologic improvement has been reported in different cases consuming such a diet.

## Additional file

Additional file 1: **Table S1.** Summary of CDS patients reported in the literature. (DOCX 30 kb)

## Abbreviations

ABHD5: α/β-hydrolase domain-containing protein 5
ALT: Alanine aminotransferase test
AST: Aspartate aminotransferase test
ATGL: Adipose triglyceride lipase
CDS: Chanarin-Dorfman syndrome
CK: Creatine kinase
GGT: Gamma glutamyl transpeptidase
LD: Lipid droplet
MCT: Medium chain triglycerides
NCIE: Non-bullous congenital ichthyosiform erythroderma
NLSDI: Neutral lipid storage disease with ichthyosis
TG: Triacylglicerol
