# Supplementary material for: Clinical and genetic characterization of a Chanarin Dorfman Syndrome patient born to diseased parents
Source: BMC Med Genet. 2018 May 29;19:88. doi: 10.1186/s12881-018-0610-0 (PMC5975656; doi:10.1186/s12881-018-0610-0)
Supplement: Supplementary file 1 — Table S1. Summary of CDS patients reported in the literature. (DOCX 30 kb) [file 12881_2018_610_MOESM1_ESM.docx]

**Table S1** Summary of CDS patients reported in the literature

| Family | Number of patients | *ABHD5* Mutation | Reference |
| --- | --- | --- | --- |
| 1 | 2 | ND | Rozenszajn, 1966; Dorfman et al, 1974 |
| 2 | 1 | ND | Dorfman et al, 1974 |
| 3 | 1 | ND |  |
| 4 | 1 | ND | Chanarin et al, 1975 |
| 5 | 1 | c.98C>G | Miranda et al, 1979; Bruno et al, 2008 |
| 6 | 4 | ND | Dykes et al, 1979 |
| 7 | 1 | c.98C>G/  c.890C>T | Angelini et al, 1980; Bruno et al, 2008 |
| 8 | 3 | c.960+5G>A | Elias et al, 1985; Redaelli et al, 2010 |
| 9 | 1 | ND | Elias et al, 1985 |
| 10 | 4 | ND | Williams et al, 1985 |
| 11 | 2 | ND | Srebrik et al, 1987; Bergman et al, 1991 |
| 12 | 1 | c.507-1 G>A | Musumeci et al, 1988 ; Bruno et al, 2008 |
| 13 | 1 | ND | Venencie et al, 1988 |
| 14 | 1 | ND | Nanda et al, 1990 |
| 15 | 2 | ND | Venencie et al, 1993 |
| 16 | 1 | ND | Dursum et al, 1993 |
| 17 | 1 | ND | Banuls et al, 1994 |
| 18 | 2 | c.898_*320del662 / c.1330_773+46del | Kakorou et al, 1997; Redaelli et al, 2010 |
| 19 | 1 | ND | Srebrink et al, 1998 |
| 20 | 1 | ND |  |
| 21 | 1 | ND | Kaassis et al, 1998 |
| 22 | 1 | ND | Gurakan et al, 1999 |
| 23 | 2 | ND | Tullu et al, 2000 |
| 24 | 1 | ND |  |
| 25 | 1 | ND | Pena-penabad et al, 2001 |
| 26 | 2 | c.19G>A | Lefèvre et al, 2001 |
| 27 | 1 | c.773-1 G>A |  |
| 28 | 1 | c.98 G>C |  |
| 29 | 1 | c.778 G>A |  |
| 30 | 1 | c.389 A>C | Lefèvre et al, 2001 |
| 31 | 1 | c.773-1 G>A |  |
| 32 | 2 | c.135-2A>G |  |
| 33 | 1 | c.46/47delAG |  |
| 34 | 3 | c.594insC |  |
| 35 | 1 | c.550C>T | Akiyama et al, 2003; Takeda et al, 2010 |
| 36 | 1 | c.700C>T | El-Kabbani et al, 2003; Redaelli et al, 2010 |
| 37 | 1 | c.752A>C | Srinivasan et al, 2004 |
| 38 | 1 | c.245A>G | Schleinitz et al, 2005 |
| 39 | 1 | c.943C>T /  c.616insGGGdel31bp | Pujol et al, 2005 |
| 40 | 1 | c.343A>G | Selma et al, 2007 |
| 41 | 1 | IVS6+6 A>T | Badeloe et al, 2008 |
| 42 | 2 | c.552G>A | Bruno et al, 2008 |
| 43 | 1 | c.552G>A |  |
| 44 | 1 | c.673delG |  |
| 45 | 1 | ND | Aksu et al, 2008 |
| 46 | 1 | c.507-1G>A | Gaeta et al, 2008 |
| 47 | 1 | ND | Pahwa et al, 2008 |
| 48 | 2 | ND | Selimoglu et al, 2009 |
| 49 | 1 | c.215T>C | Ujihara et al, 2010 |
| 50 | 1 | c.150C>G /  c.217T>G | Emre et al, 2010 |
| 51 | 1 | c.150C>G /  c.217T>G |  |
| 52 | 1 | c.594insC |  |
| 53 | 1 | c.594insC |  |
| 54 | 1 | c.594insC |  |
| 55 | 1 | c.594insC |  |
| 56 | 1 | IVS1+1G>A | Cakir et al, 2010 |
| 57 | 1 | c.47+1G>A | Redaelli et al, 2010 |
| 58 | 2 | c.960+5G>A |  |
| 59 | 1 | c.898_*320del |  |
| 60 | 1 | ND | Mitra et al, 2010 |
| 61 | 1 | c.700C>T | Pike et al, 2011 |
| 62 | 2 | ND | Chander et al, 2011 |
| 63 | 1 | c.960+5G>A | Israeli et al, 2011 |
| 64 | 4 | c.506ins101 bp | Samuelov et al, 2011 |
| 65 | 3 | c.1006G>T | Aggarwal et al, 2012 |
| 66 | 2 | c.594insC | Cakmak et al, 2012 |
| 67 | 1 | c.943C>T | Elitzur et al, 2012 |
| 68 | 1 | ND | Chilkar et al, 2012 |
| 69 | 1 | ND | Ersoy et al, 2012 |
| 70 | 1 | ND | Singh et al, 2012 |
| 71 | 1 | ND |  |
| 72 | 1 | ND |  |
| 73 | 2 | c.594insC | Camlar et al, 2013 |
| 74 | 1 | c.506-3C>G | Srinivasaraghavan et al, 2014 |
| 75 | 1 | g.43728907_43732862del3955ins26 | Missaglia et al, 2014 |
| 76 | 2 | c.773-1G>A | Sugiura et al, 2014 |
| 77 | 3 | c.594insC | Gomez-Moyano et al, 2014 |
| 78 | 1 | c.773+1delG | Tamhankar et al, 2014 |
| 79 | 1 | c.47+1G>A | Huigen et al, 2014 |
| 80 | 1 | c.594insC |  |
| 81 | 4 | c.594insC | Nur et al, 2015 |
| 82 | 1 | ND | Kazemi et al, 2015 |
| 83 | 1 | ND | Mogahed et al, 2015 |
| 84 | 1 | c.594insC | Barnerias et al, 2015 |
| 85 | 1 | c.413G>A | Unlusoy et al, 2015 |
| 86 | 1 | c.297C>A | Gupta et al, 2016 |
| 87 | 1 | ND | Ameur et al, 2016 |
| 88 | 1 | c.700C>T /  c.838C>T | Takeity et al, 2016 |
| 89 | 1 | ND | Waheed et al, 2016 |
| 90 | 1 | ND | Arora et al, 2017 |
| 91 | 1 | c.594dupC | Demir et al, 2017 |
| 92 | 1 | c.752A>C | Verma et al, 2017 |
| 93 | 2 | c. 560_578del19bp | Nakhaei et al, 2018 |
